# Frontotemporal dementia: Clinical aspects, genetics, and neuropathology of a family with a *C9ORF72* expansion in Argentina

**DOI:** 10.1111/bpa.70057

**Published:** 2026-01-07

**Authors:** Karen Daniela Román, Carolina Agata Ardohain, Ezequiel I. Surace, Mónica Beatriz Mezmezian, Alejandro Levy, Alice Baez Lovera, Carlos Turizo, Marcos G. Sorbara, María M. Esnaola y Rojas, Gastón H. Graviotto, Gustavo Sevlever, Ricardo F. Allegri, Cecilia M. Serrano, Nahuel Magrath Guimet

**Affiliations:** ^1^ Cognitive Neurology Department Unidad Asistencial Dr. César Milstein Ciudad Autónoma de Buenos Aires Argentina; ^2^ Neurology Translational Medicine Unit, Cognitive Impairment Laboratory of the “Eva Perón” Hospital Ciudad Autónoma de Buenos Aires Argentina; ^3^ Cognitive Neurology, Neuropsychology and Neuropsychiatry Department FLENI Ciudad Autónoma de Buenos Aires Argentina; ^4^ Laboratory of Neurodegenerative Diseases Institute of Neurosciences‐FLENI (CONICET) Ciudad Autónoma de Buenos Aires Argentina; ^5^ Brain Bank, Neuropathology and Molecular Biology Laboratory FLEN Ciudad Autónoma de Buenos Aires Argentina; ^6^ Neurology Center for Medical Education and Clinical Research (CEMIC) Ciudad Autónoma de Buenos Aires Argentina; ^7^ Neurology Central Nervous System, IQVIA Ciudad Autónoma de Buenos Aires Argentina; ^8^ Neurology Atlantic Fellow for Equity in Brain Health, Global Brain Health Institute, University of California San Francisco (UCSF) San Francisco California USA

**Keywords:** C9ORF72 expansion, cognitive neurology, familial neurodegenerative disease, frontotemporal dementia, frontotemporal lobar degeneration, genetics of dementia, neuropathology

## Abstract

Frontotemporal dementia (FTD) is the second most common cause of early‐onset dementia, typically manifesting before the age of 65, with a mean onset at 58 years. FTD may encompass a spectrum of neurodegenerative disorders resulting from frontotemporal lobar degeneration (FTLD), affecting behavior, language, and motor function. Among its clinical variants, the behavioral variant (bvFTD) is the most frequently inherited, often associated with mutations in MAPT, GRN, and C9ORF72, the latter being the most prevalent genetic cause of FTD and FTD‐motor neuron disease (FTD‐MND). While bvFTD is classically defined by profound behavioral changes and executive dysfunction, cases linked to C9ORF72 expansions exhibit atypical neuropsychiatric features. This study documents two cases within the same family presenting with bvFTD and atypical parkinsonism, associated with a C9ORF72 expansion. Neurocognitive assessments, genetic testing, and neuroimaging (MRI, SPECT) were performed to characterize the clinical phenotype. A detailed review of the familial aggregation of neurodegenerative and psychiatric disorders provided further insight into the genetic contributions to symptomatology. The findings highlight the phenotypic heterogeneity associated with C9ORF72 expansions, demonstrating a spectrum ranging from bvFTD to atypical parkinsonism, with variable neuropsychiatric involvement. While movement disorders in FTD have historically been underestimated, these cases reinforce the association between parkinsonism and familial bvFTD. Given the limited epidemiological data on genetic FTD in Latin America, this study underscores the importance of genetic testing in cases with prominent behavioral and psychiatric symptoms, supporting early identification and genetic counseling for affected families.

## Graphical abstract

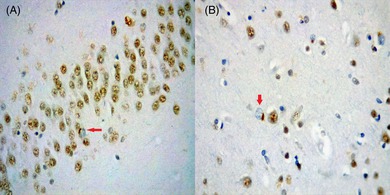

## 1 INTRODUCTION

Frontotemporal dementia (FTD) is the second most common cause of early‐onset dementia, typically manifesting before the age of 65, with a mean onset around 58 years [1]. The clinical syndrome of FTD, characterized by behavioral, speech or language dysfunction, and motor symptoms, may result from a range of underlying pathologies, most commonly frontotemporal lobar degeneration (FTLD). Pathologic FTLD subtypes include FTLD‐TDP, FTLD‐tau, FTLD‐FUS, and other rarer forms. Approximately 20%–25% of individuals with FTLD are estimated to carry a mutation associated with a specific FTLD pathology [2].

FTD manifests in multiple clinical variants: the behavioral variant (bvFTD), the semantic and non‐fluent variants of primary progressive aphasia (svPPA and nfvPPA), and FTD associated with motor neuron disease (FTD‐MND) [3, 4]. Additionally, bvFTD is a common clinical presentation of 4R tauopathy subtypes, including progressive supranuclear palsy (PSP), corticobasal degeneration (CBD), and argyrophilic grain disease (AGD). PSP pathology accounts for approximately 5% of bvFTD cases, while TDP‐43 pathology is found in about 50%, underscoring the heterogeneity of the underlying proteinopathies [5].

Approximately 40% of FTD cases have a family history, though only about 20% exhibit a clear autosomal dominant inheritance pattern [6]. Among the clinical variants, the behavioral variant is the most frequently inherited [7]. In roughly one‐third of cases, FTD is linked to genetic mutations, including *MAPT*, *GRN*, or *C9ORF72*. The *C9ORF72* mutation is the most common genetic cause of FTD and FTD‐MND [6, 8]. The onset of symptoms in *C9ORF72* mutation carriers ranges from 27 to 83 years, with a median age of around 50 years [9]. Median survival is approximately 6–11 years from symptom onset and 3–4 years from diagnosis [4]. Mutations in “FTD genes,” including *GRN*, *MAPT*, and *C9ORF72*, may present with a clinical phenotype that is indistinguishable from typical Alzheimer's disease (AD); this fact has important implications for clinicians, who should consider both “FTD” and “AD” genes when evaluating families with strong histories of AD [10], underscoring the critical role of genetic testing in achieving diagnostic accuracy.

The clinical presentations linked to *C9ORF72* mutations primarily manifest as the typical bvFTD [11, 12]. However, unlike the classical variant, psychiatric symptoms such as delusions and hallucinations have been documented in up to 40% of patients with this mutation [13]. Other associated features include abnormal movements, parkinsonism, and motor neuron disease. Parkinsonism is present in approximately 20%–30% of patients with FTLD and may appear across all FTLD subtypes. Some individuals with familial or sporadic FTLD present with prominent parkinsonism, typically of the akinetic‐rigid subtype [14].

There is limited epidemiological information on genetic mutations associated with FTD in our region. Our report may contribute to raising clinical suspicion and promoting genetic testing in other cases.

## 2 CASE 1

The proband, a woman with a history of hypertension, hypothyroidism, and former tobacco use, was referred to neurology for evaluation of a 4‐year history of parkinsonism and progressive cognitive decline. At age 63, she initially consulted a psychiatrist due to mood disturbances, interpreted as a depressive syndrome, and was treated with duloxetine (30 mg/day) and mirtazapine (30 mg/day) with minimal clinical response. One year later, she developed worsening gait slowness and postural instability, resulting in recurrent falls. A general practitioner initiated pramipexole (0.75 mg three times daily), without significant improvement.

**FIGURE 1:**
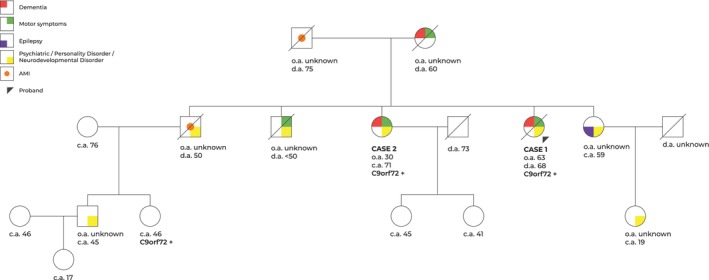
Patient's family medical history pedigree. The pedigree illustrates a multigenerational family history characterized by a broad spectrum of clinical conditions. Standard pedigree symbols are used: Squares for males, circles for females, with distinct colors used to denote specific clinical features. The proband, indicated by a black arrow, corresponds to Case 1. The family history includes early‐onset dementia, psychiatric and behavioral disturbances, personality disorders, neurodevelopmental disorders, motor symptoms, epilepsy, and acute myocardial infarction (AMI). AMI, acute myocardial infarction; psychiatric, psychiatric symptoms; C9orf72+, tested positive for the C9orf72 hexanucleotide repeat expansion; o.a: age of onset; c.a, current age; d.a, age of death.

She belonged to a multigenerational family with a complex clinical history, as detailed in the pedigree (Figure 1). Reported conditions include early‐onset dementia, psychiatric, neurological, and personality disorders, motor symptoms, epilepsy, and acute myocardial infarction.

Prior neuropsychological evaluation of Case 1 revealed deficits in memory, attention, and executive functioning. Although a formal assessment of social cognition could not be performed, her informant reported longstanding empathy deficits and socially disruptive behaviors. The brain MRI showed signs of diffuse brain atrophy with left frontotemporal predominance. Cerebral SPECT revealed hypoperfusion in the orbitofrontal, anterior prefrontal, and dorsolateral cortex, with a slight left‐sided predominance. Additionally, decreased perfusion was observed in the left inferior frontal gyrus, left superior premotor cortex, bilateral striatum (more pronounced on the left), bilateral thalami, and insular cortex.

Given the symptoms consistent with familial aggregation (Goldman score of 1 and “High” Category according to the Criteria for FTLD Spectrum Disorder Pedigree Categorization) and the presence of hallucinations in one of her sisters (Case 2), previously reported in cases of *C9ORF72* gene mutations, we opted for single‐gene testing. This testing identified an abnormal expansion (over 30 repeats) of the GGGGCC hexanucleotide in the *C9ORF72* gene (genotype 2) in Case 1.

**FIGURE 2:**
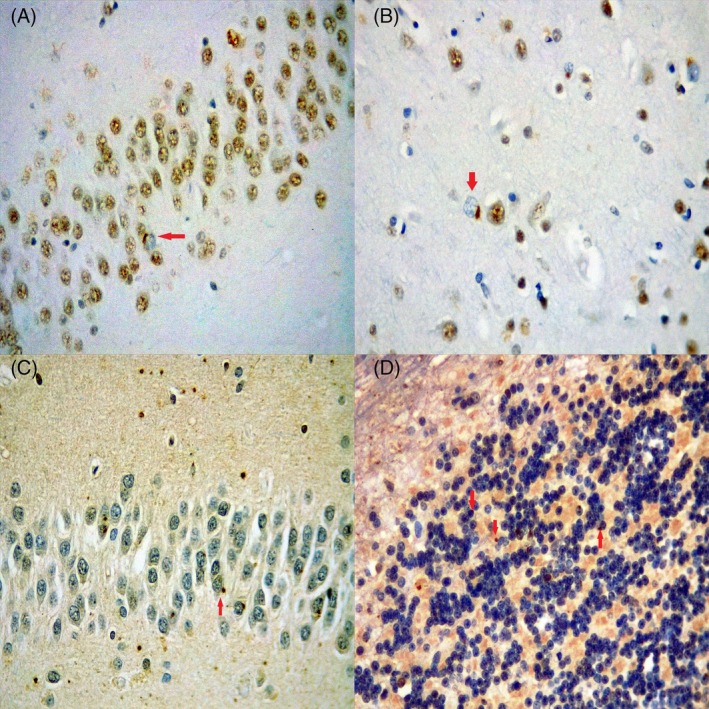
Histopathological findings in brain tissue from Case 1: (A) and (B) correspond to immunohistochemistry for TDP‐43: (A)—Dentate gyrus; (B)—Temporal lobe. (C) and (D) correspond to immunohistochemistry for ubiquitin: (C)—Dentate gyrus; (D)—Granular layer of the cerebellum. In all four images, red arrows indicate neuronal cytoplasmic inclusions.

The patient died at the age of 68, and a post‐mortem pathological examination revealed a reduction in the number of neurons in the superficial layers of the cortex, along with mild spongiosis in the frontal and temporal lobes. TDP‐43 immunostaining revealed dystrophic neurites and isolated neuronal cytoplasmic inclusions in the amygdala, dentate gyrus (Figure 2A), superficial layers of the frontal and temporal lobes (Figure 2B), and basal ganglia. Immunohistochemistry for ubiquitin revealed isolated neuronal cytoplasmic inclusions in the superficial layers of the frontal and temporal lobes, the dentate gyrus (Figure 2C), pyramidal cells of the hippocampus, the basal ganglia, and the granular layer of the cerebellum (Figure 2D). A diagnosis of FTLD‐TDP Type A was made. Additionally, immunohistochemistry using the Tau PHF‐1 antibody revealed grains and pretangles with a perinuclear halo in the CA2 region of the hippocampus, features characteristic of AGD, a common tauopathy observed in older patients. These AGD findings are likely unrelated to the patient's genetic results.

## 3 CASE 2

The older sister of the proband (Figure 1) had a history of hypertension, diabetes mellitus, dyslipidemia, and was a former smoker as well. She started at the age of 30 years with behavioral disturbances and a lack of personal hygiene and was diagnosed with bipolar disorder. She was initially diagnosed with early‐onset Lewy body dementia years before our evaluation due to progressive memory decline, hallucinations, and parkinsonism, which progressed to clinical dementia in approximately 4 years, ultimately requiring institutionalization. Her neuropsychological assessment revealed a multidomain cognitive impairment. Based on the clinical manifestations and known family history, genetic testing for *C9ORF72* mutation was performed at the age of 70, confirming the presence of a pathogenic GGGGCC hexanucleotide expansion in the *C9ORF72* gene. Neuropathological data are not available for Case 2, as the individual is still alive.

Only Cases 1 and 2 underwent formal evaluation by the clinical team. The pedigree (Figure 1) was compiled based on detailed and structured information provided by one of the daughters of Case 2, who served as the primary informant. According to her account, other family members presented with various neuropsychiatric conditions: one niece had a diagnosis of attention‐deficit hyperactivity disorder (ADHD), a nephew was diagnosed with obsessive‐compulsive disorder, and substance abuse was reported in one of the brothers. Additionally, unspecified personality disorders were noted in other relatives. Although these conditions differ in clinical classification, they were grouped under a single category in the pedigree (Figure 1) to denote the presence of any diagnosis within the broader neuropsychiatric spectrum. This approach was adopted to streamline the visual representation.

Motor symptoms were reported in the mother and one brother of Cases 1 and 2, presenting as progressive gait impairment and dysphagia, ultimately leading to wheelchair dependence. Although no definitive neurological diagnosis was established in these individuals, the clinical features raise the possibility of an underlying motor neuron disease; however, limited clinical data preclude a conclusive interpretation. In contrast, motor manifestations in Cases 1 and 2 were consistent with atypical parkinsonism, with no clinical or electromyographic evidence of motor neuron disease. Both tested positive for the C9orf72 hexanucleotide repeat expansion.

An asymptomatic niece, evaluated independently in a separate clinic abroad, was also found to carry the C9orf72 expansion, despite the absence of clinical symptoms at the time of assessment.

Several other family members were not clinically or genetically assessed due to factors such as prior death, lack of interest in evaluation, logistical constraints limiting access to testing. A few assessments are currently underway. These limitations should be considered when interpreting the inheritance pattern and penetrance of the C9orf72 mutation within the family.

## 4 DISCUSSION

The first report on the frequency of the *C9ORF72* expansion in a Latin American population found that the G4C2 expansion frequency was similar to that reported for patients in Europe and North America, while the frequency in the sporadic amyotrophic lateral sclerosis (ALS) group was significantly lower [15]. The first reported case of FTD associated with a *C9ORF72* mutation in Argentina, which involved a family history of ALS and parkinsonism, also informed the presence of a G4C2 hexanucleotide repeat expansion in the *C9ORF72* gene [16]. These observations are partially reflected in the family described in our study. Electromyographic studies were performed in Cases 1 and 2 to evaluate for motor neuron disease; however, the findings were not compatible with this diagnosis despite the presence of a G4C2 hexanucleotide repeat expansion in *C9ORF72*. However, other family members exhibited unspecified motor symptoms, such as progressive gait impairment and dysphagia, but could not be formally assessed. These limitations hinder a reliable estimation of the true extent of MND manifestations within the reported family. Further investigation of descendants is currently underway.

Individuals with *C9ORF72* expansions may present atypical neuropsychiatric manifestations of bvFTD, including hallucinations or delusions. Importantly, family members of *C9ORF72* carriers face an increased risk of psychiatric disorders, such as autism spectrum disorders, psychotic illnesses like schizophrenia, mood disorders, and suicide [17]. In our documented cases, the family exhibited multiple neuropsychiatric symptoms that were initially misinterpreted as primary psychiatric disorders for several years before the genetic diagnosis of the index case. We found other studies that describe similar manifestations in individuals with *C9ORF72* expansion. One study involving 648 FTD patients compared individuals carrying mutations in *C9ORF72* with non‐carriers, revealing that delusions were the only symptom more frequently reported at presentation among bvFTD mutation carriers [18]. This was corroborated by another study involving 144 FTD patients, where mutation carriers were similarly more prone to psychotic symptoms [13]. Relative sparing of episodic memory is a diagnostic criterion of behavioral variant frontotemporal dementia (bvFTD), but increasing evidence suggests that bvFTD patients can show episodic memory deficits at a similar level as AD [19, 20], this was also seen in our index case.

Movement disorders are frequent in *C9ORF72* expansions. They may precede signs of ALS or frontotemporal dementia, or even be present in isolation [21]. The atypical parkinsonism exhibited by Case 1 and 2, coupled with the neuropsychiatric symptoms and predominantly executive cognitive impairment, led us to suspect a familial form of FTD. While other conditions associated with parkinsonism and cognitive impairment could have been considered, such as Lewy Body dementia, the familial aggregation raised the suspicion of a genetic condition. FTD associated with the *C9ORF72* mutation explained the manifestations observed in the patient and her family. Parkinsonism is the most common movement disorder in patients with bvFTD [3], typically presenting as a symmetrical akinetic‐rigid syndrome with gait disturbance, with or without tremor, usually postural or action‐related, and rarely resting. The high frequency of parkinsonism in FTD patients led once to the inclusion of akinesia, rigidity, and tremor in the supportive diagnostic features of the 1998 FTD diagnostic criteria. However, these physical features were excluded from the 2011 consensus diagnostic criteria, which focus on cognitive and behavioral symptoms [17].

Notable intrafamilial heterogeneity in clinical presentation has been documented, including variability in symptom onset, progression, and associated motor features [22]. *C9ORF72* expansions may present as a slowly progressive behavioral variant of frontotemporal dementia (bvFTD‐SP) and should be considered in patients with this phenotype, particularly when there is a family history of dementia and/or motor neuron disease [23], psychosis, including visual and auditory hallucinations and delusions [24], and other movement disorders such as parkinsonism [21].

To assess the likelihood of a pathogenic mutation based on family history, the modified Goldman score provides a structured framework. This scoring system stratifies familial risk: a score of 1 reflects an autosomal dominant inheritance pattern with at least three affected individuals across two generations; score 2 indicates familial clustering of three or more affected individuals without meeting criteria for score 1; score 3 denotes a single affected relative (with subcategories based on age of onset); and score 4 corresponds to the absence of a known family history of neurodegenerative disease [25]. This tool is particularly useful for guiding decisions about genetic testing in resource‐limited settings.

Complementary to this, the Criteria for FTLD Spectrum Disorder Pedigree Categorization further refines the probability of a pathogenic mutation specifically in FTD. Families are classified into five categories—high, medium, low, apparent sporadic, and unknown significance—based on the number and degree of affected relatives with FTLD‐spectrum or other neurodegenerative disorders [26].

According to the available literature, the progression of *C9ORF72*‐associated FTD is neuroanatomically linked to diffuse and variable cortical and central atrophy, with more consistent involvement of the cerebellum and thalamus [20]. These findings were not observed on MRI in our cases; however, thalamic hypoperfusion was detected on the brain SPECT of the index case.

To our knowledge, this is the first case reported with histopathological data in ours country. Regarding the findings in the post‐mortem samples from the index case, the autopsy revealed FTLD‐TDP Type A, with no pathological changes observed at the cervical spinal cord level. Beyond the histopathological findings, the clinical phenotype of the proband is consistent with that reported in the literature for FTLD‐TDP Type A. Most cases of FTLD‐TDP Type A present with features of behavioral variant frontotemporal dementia (bvFTD), often characterized by prominent apathy and social withdrawal. Executive dysfunction and some degree of memory impairment are not uncommon, particularly in individuals with older age at onset. Extrapyramidal features are reported in up to half of cases but are rarely the presenting or predominant symptom, whereas ALS is highly unusual [27].

Additionally, immunohistochemical changes consistent with AGD were identified in Case 1; this is a common age‐related tauopathy observed across a range of neurodegenerative disorders. The contribution of AGD to the clinical phenotype remains controversial, as it often occurs with co‐morbid pathologies and has been reported in up to 30% of people with no known cognitive impairment [28]. There is no known pathogenic link between AGD and *C9ORF72* expansions [29]. While the presence of argyrophilic grains may not be directly relevant to the genetic focus of this report, it reflects the frequent coexistence of age‐associated proteinopathies in postmortem brain tissue. Although mixed pathologies are typically observed in older adults, our case involved symptom onset before 65 years of age, a context in which single neurodegenerative processes are more commonly expected [30, 31]. These findings reinforce the value of postmortem studies in capturing the full spectrum of neuropathological changes, even when their clinical relevance may be limited.

Despite the lack of a current treatment for this disease, genetic testing and histopathological findings offer valuable insights that may help identify future treatment targets. This underscores the importance of early detection and considering these disorders in clinical management. Early diagnosis not only enables genetic counseling for asymptomatic individuals but also opens the possibility for participation in future clinical trials, particularly given the absence of effective treatments at present.

## AUTHOR CONTRIBUTIONS

All authors were involved in the study conception and design. The first draft of the manuscript was prepared by Dr. Karen D. Román and Dr. Carolina A. Ardohain, while the figures were created by Dr. Mónica Beatriz Mezmezian and Dr. Alejandro Levy. All authors reviewed and provided feedback on previous versions of the manuscript and approved its final version.

## FUNDING INFORMATION

No funding was received for this study.

## CONFLICT OF INTEREST STATEMENT

The authors declare no conflicts of interest.

## ETHICS STATEMENT

The study was approved by the Bioethics Committee of Unidad Asistencial Dr. César Milstein, with consent obtained from both patients and their families.

## INFORMED CONSENT

All participants provided informed consent.

## Data Availability

The data that support the findings of this study are available from the corresponding author upon reasonable request.
